# Supplementary material for: Does Music Therapy Improve Gait after Traumatic Brain Injury and Spinal Cord Injury? A Mini Systematic Review and Meta-Analysis
Source: Brain Sci. 2023 Mar 21;13(3):522. doi: 10.3390/brainsci13030522 (PMC10046548; doi:10.3390/brainsci13030522)
Supplement: Supplementary file 1 [file brainsci-13-00522-s001.zip › brainsci-2253516-supplementary.pdf]

Does music therapy improve gait after traumatic brain injury and spinal cord injury? A mini systematic review and meta-analysis

Shashank Ghai

Table S1. PRISMA 2020 Checklist

Section and Topic	Item #	Checklist item	Location where item is reported
**TITLE**			
Title	1	Identify the report as a systematic review.	1
**ABSTRACT**			
Abstract	2	See the PRISMA 2020 for Abstracts checklist.	1
**INTRODUCTION**			
Rationale	3	Describe the rationale for the review in the context of existing knowledge.	1-3
Objectives	4	Provide an explicit statement of the objective(s) or question(s) the review addresses.	3
**METHODS**			
Eligibility criteria	5	Specify the inclusion and exclusion criteria for the review and how studies were grouped for the syntheses.	3
Information sources	6	Specify all databases, registers, websites, organisations, reference lists and other sources searched or consulted to identify studies. Specify the date when each source was last searched or consulted.	3
Search strategy	7	Present the full search strategies for all databases, registers and websites, including any filters and limits used.	3, Supplementary table 2
Selection process	8	Specify the methods used to decide whether a study met the inclusion criteria of the review, including how many reviewers screened each record and each report retrieved, whether they worked independently, and if applicable, details of automation tools used in the process.	3
Data collection process	9	Specify the methods used to collect data from reports, including how many reviewers collected data from each report, whether they worked independently, any processes for obtaining or confirming data from study investigators, and if applicable, details of automation tools used in the process.	3
Data items	10a	List and define all outcomes for which data were sought. Specify whether all results that were compatible with each outcome domain in each study were sought (e.g. for all measures, time points, analyses), and if not, the methods used to decide which results to collect.	3-4
	10b	List and define all other variables for which data were sought (e.g. participant and intervention characteristics, funding sources). Describe any assumptions made about any missing or unclear information.	4
Study risk of bias assessment	11	Specify the methods used to assess risk of bias in the included studies, including details of the tool(s) used, how many reviewers assessed each study and whether they worked independently, and if applicable, details of automation tools used in the process.	4
Effect measures	12	Specify for each outcome the effect measure(s) (e.g. risk ratio, mean difference) used in the synthesis or presentation of results.	4
Synthesis methods	13a	Describe the processes used to decide which studies were eligible for each synthesis (e.g. tabulating the study intervention characteristics and comparing against the planned groups for each synthesis (item #5)).	4
	13b	Describe any methods required to prepare the data for presentation or synthesis, such as handling of missing summary statistics, or data conversions.	4

Section and Topic	Item #	Checklist item	Location where item is reported
	13c	Describe any methods used to tabulate or visually display results of individual studies and syntheses.	4
	13d	Describe any methods used to synthesize results and provide a rationale for the choice(s). If meta-analysis was performed, describe the model(s), method(s) to identify the presence and extent of statistical heterogeneity, and software package(s) used.	4
	13e	Describe any methods used to explore possible causes of heterogeneity among study results (e.g. subgroup analysis, meta-regression).	4
	13f	Describe any sensitivity analyses conducted to assess robustness of the synthesized results.	4
Reporting bias assessment	14	Describe any methods used to assess risk of bias due to missing results in a synthesis (arising from reporting biases).	4
Certainty assessment	15	Describe any methods used to assess certainty (or confidence) in the body of evidence for an outcome.	4
**RESULTS**			
Study selection	16a	Describe the results of the search and selection process, from the number of records identified in the search to the number of studies included in the review, ideally using a flow diagram.	4-5, Figure 1
	16b	Cite studies that might appear to meet the inclusion criteria, but which were excluded, and explain why they were excluded.	Figure 1
Study characteristics	17	Cite each included study and present its characteristics.	5-10, Table 1 and 2
Risk of bias in studies	18	Present assessments of risk of bias for each included study.	11, Figure 2, Table 3
Results of individual studies	19	For all outcomes, present, for each study: (a) summary statistics for each group (where appropriate) and (b) an effect estimate and its precision (e.g. confidence/credible interval), ideally using structured tables or plots.	14-15
Results of syntheses	20a	For each synthesis, briefly summarise the characteristics and risk of bias among contributing studies.	Table 2
	20b	Present results of all statistical syntheses conducted. If meta-analysis was done, present for each the summary estimate and its precision (e.g. confidence/credible interval) and measures of statistical heterogeneity. If comparing groups, describe the direction of the effect.	16, Table 3 and 4
	20c	Present results of all investigations of possible causes of heterogeneity among study results.	21, Table 5
	20d	Present results of all sensitivity analyses conducted to assess the robustness of the synthesized results.	21, Table 5
Reporting biases	21	Present assessments of risk of bias due to missing results (arising from reporting biases) for each synthesis assessed.	14, Figure 3
Certainty of evidence	22	Present assessments of certainty (or confidence) in the body of evidence for each outcome assessed.	16, Table 3, 4, and 5
**DISCUSSION**			
Discussion	23a	Provide a general interpretation of the results in the context of other evidence.	22-25
	23b	Discuss any limitations of the evidence included in the review.	22-23

Section and Topic	Item #	Checklist item	Location where item is reported
	23c	Discuss any limitations of the review processes used.	24
	23d	Discuss implications of the results for practice, policy, and future research.	24-25
**OTHER INFORMATION**			
Registration and protocol	24a	Provide registration information for the review, including register name and registration number, or state that the review was not registered.	3 (https://osf.io/xb8zn)
	24b	Indicate where the review protocol can be accessed, or state that a protocol was not prepared.	3
	24c	Describe and explain any amendments to information provided at registration or in the protocol.	-
Support	25	Describe sources of financial or non-financial support for the review, and the role of the funders or sponsors in the review.	25
Competing interests	26	Declare any competing interests of review authors.	25
Availability of data, code and other materials	27	Report which of the following are publicly available and where they can be found: template data collection forms; data extracted from included studies; data used for all analyses; analytic code; any other materials used in the review.	25

From: Page MJ, McKenzie JE, Bossuyt PM, Boutron I, Hoffmann TC, Mulrow CD, et al. The PRISMA 2020 statement: an updated guideline for reporting systematic reviews. BMJ 2021;372:n71. doi: 10.1136/bmj.n71

Table S2. Search keywords

PICOS	Strategy	Terms
**P**	**#1**	(‘TBI’ OR ‘TBIs’ OR ‘Traumatic brain injury’ OR ‘Traumatic Brain Injuries’ OR ‘Trauma’ OR ‘Brain’ OR ‘Brain Injuries’ OR ‘Head Injuries’ OR ‘Closed Head Injuries’ OR ‘Penetrating’ OR ‘Brain Injuries’ OR ‘Traumatic’ OR ‘Concussion’ OR ‘Brain’ OR ‘Head Injury’ OR ‘Penetrating’ OR ‘Head Injury, Closed’ OR ‘Trauma’ OR ‘Nervous System’ OR ‘Brain Concussion’ OR ‘Traumatic Encephalopathy’ OR ‘Trauma’ OR ‘Brain’ OR ‘Brain Injuries’)/de OR (TBI OR TBIs OR Traumatic brain injury OR Traumatic Brain Injuries OR Trauma OR Brain OR Brain Injuries OR Head Injuries OR Closed Head Injuries OR Penetrating OR Brain Injuries OR Traumatic OR Concussion OR Brain OR Head Injury OR Penetrating OR Head Injury, Closed OR Trauma OR Nervous System OR Brain Concussion OR Traumatic Encephalopathy OR Trauma OR Brain OR Brain Injuries);ti;ab
		(‘SCI’ OR ‘SCIs’ OR ‘Spinal cord injury’ OR ‘Spinal cord injuries’ OR ‘Injuries’ OR ‘Spinal Cord’ OR ‘Myelopathy’ OR ‘Traumatic’ OR ‘Post-Traumatic Myelopathy’ OR ‘Spinal Cord Contusion’ OR ‘Spinal Cord Laceration’ OR ‘Spinal Cord Transection’ OR ‘Spinal Cord Trauma’ OR ‘Paraplegia’ OR ‘Quadriplegia’)/de OR (SCI OR SCIs OR Spinal cord injury OR Spinal cord injuries OR Injuries OR Spinal Cord OR Myelopathy OR Traumatic OR Post-Traumatic Myelopathy OR Spinal Cord Contusion OR Spinal Cord Laceration OR Spinal Cord Transection OR Spinal Cord Trauma OR Paraplegia OR Quadriplegia);ti;ab
		((‘age groups’ OR ‘adolescent’ OR ‘young’ OR ‘elderly’ OR ‘old’)/de OR ((age groups OR adolescent OR young OR elderly OR old OR);ti;ab
**I**	**#2**	(“rhythmic auditory cueing” OR “rhythmic acoustic cueing” OR “rhythmic auditory entrainment” OR “metronome cueing” OR “metronome” OR “rhythmic metronome cueing” OR “acoustic stimulus” OR “acoustic cueing” OR “acoustic cueing” OR “external stimuli” OR “external cueing” OR “external cueing” OR “music therapy” OR “Neurological music therapy” OR “tempo” OR “beat” OR “rhythm” OR “RAC” OR “NMT”)/de OR (rhythmic auditory cueing OR rhythmic auditory cueing OR rhythmic acoustic cueing OR rhythmic auditory entrainment OR metronome cueing OR metronome OR rhythmic metronome cueing OR acoustic stimulus OR acoustic cueing OR acoustic cueing OR external stimuli OR external cueing OR external cueing OR music therapy OR Neurological music therapy OR tempo OR beat OR rhythm OR RAC OR NMT)ti,ab
**C**	**#3**	'Physiotherapy' OR 'Conventional therapy' OR 'Rehab' OR 'Rehabilitation' OR 'Physical rehabilitation' OR 'Physical therapy' OR 'Gait therapy')/de OR (Physiotherapy OR Conventional therapy OR Rehab OR Rehabilitation OR Physical rehabilitation OR Physical therapy OR Gait therapy);ti;ab

**O**	**#4**	('Spatiotemporal gait outcome' OR 'Gait speed' OR 'Gait velocity' OR 'Walking speed' OR 'Walking velocity' OR 'Stride length' OR 'Step length' OR 'Stride time' OR 'Step time' OR 'Cadence' OR 'Gait outcomes' OR 'Gait deviation index' OR 'Kinematic parameters' OR 'Range of motion' OR 'Gross motor function test' OR 'Single support time' OR 'Double support time' OR 'Functional gait assessment')/de OR (Spatiotemporal gait outcome OR Gait speed OR Gait velocity OR Walking speed OR Walking velocity OR Stride length OR Step length OR Stride time OR Step time OR Cadence OR Gait outcomes OR Gait deviation index OR Kinematic parameters OR Range of motion OR Gross motor function test OR Single support time OR Double support time OR Functional gait assessment):ti,ab
**S**	**#5**	clinical trial/exp OR (‘intervention study’ OR ‘cohort analysis’ OR ‘longitudinal study’ OR ‘cluster analysis’ OR ‘crossover trial’ OR ‘cluster analysis’ OR ‘randomized trial’ OR ‘major clinical study’)/de OR (intervention study OR cohort analysis OR longitudinal study OR cluster analysis OR crossover trial OR cluster analysis OR randomized trial OR major clinical study);ti,ab

Sensitivity analysis

Study name	Subgroup within study	Point	Standard error	Variance	Lower limit	Upper limit	Z-Value	p-Value
Thompson et al. (2021)	Cadence	0.561	0.136	0.018	0.295	0.828	4.127	0.000
Park et al. (2015)	Speed	0.490	0.129	0.017	0.238	0.742	3.807	0.000
Thompson et al. (2021)	Step length	0.561	0.136	0.019	0.294	0.828	4.116	0.000
Sheridan et al. (2021)	Step length	0.547	0.135	0.018	0.283	0.812	4.054	0.000
Park et al. (2015)	Step length	0.543	0.135	0.018	0.280	0.807	4.037	0.000
Park et al. (2015)	Stride length	0.513	0.133	0.018	0.252	0.773	3.854	0.000
Wilfong (2009)	Stride length	0.521	0.137	0.019	0.253	0.790	3.809	0.000
Hurt et al. (1998)	Stride length	0.523	0.138	0.019	0.253	0.793	3.800	0.000
Park et al. (2015)	Symmetry	0.490	0.129	0.017	0.237	0.742	3.804	0.000
Hurt et al. (1998)	Symmetry	0.547	0.137	0.019	0.277	0.816	3.979	0.000
Sheridan et al. (2021)	Cadence	0.556	0.131	0.017	0.299	0.813	4.238	0.000
Park et al. (2015)	Cadence	0.516	0.134	0.018	0.254	0.779	3.859	0.000
Wilfong (2009)	Cadence	0.480	0.131	0.017	0.222	0.737	3.656	0.000
Hurt et al. (1998)	Cadence	0.539	0.138	0.019	0.268	0.809	3.904	0.000
Wilfong (2009)	Speed	0.505	0.134	0.018	0.241	0.768	3.752	0.000
Hurt et al. (1998)	Speed	0.527	0.138	0.019	0.256	0.797	3.820	0.000
Thompson et al. (2021)	Speed	0.561	0.136	0.019	0.294	0.828	4.117	0.000
Sheridan et al. (2021)	Speed	0.551	0.134	0.018	0.288	0.813	4.107	0.000
Pooled		0.526	0.128	0.017	0.274	0.778	4.092	0.000

Statistics with study removed

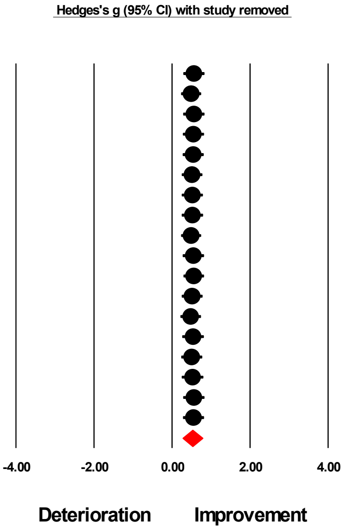

Figure S1. Forest plot depicting the results of leave-one-out sensitivity analysis for the effect of MT on overall spatiotemporal gait outcomes in people with traumatic brain injury. The plot shows the effect size estimates (circles) and their confidence intervals (horizontal lines) for each study, with the overall effect size estimate (red diamond at the bottom) and its confidence interval (diamond outline) presented. The change in the overall effect size estimate after the removal of each study is shown by a black circle presented in the right-hand column.

Study name	Point	Standard error	Variance	Lower limit	Upper limit	Z-Value	p-Value
Thompson et al. (2021)	0.836	0.412	0.169	0.030	1.643	2.032	0.042
Sheridan et al. (2021)	0.804	0.394	0.155	0.031	1.576	2.040	0.041
Park et al. (2015)	0.443	0.248	0.061	-0.043	0.928	1.787	0.074
Wilfong (2009)	0.608	0.411	0.169	-0.197	1.413	1.481	0.139
Hurt et al. (1998)	0.716	0.440	0.194	-0.147	1.579	1.626	0.104
Pooled	0.643	0.323	0.104	0.011	1.275	1.993	0.046

Statistics with study removed

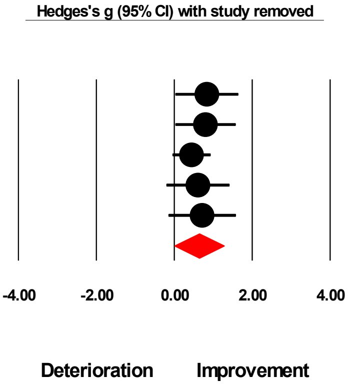

Figure S2. Forest plot depicting the results of leave-one-out sensitivity analysis for the effect of MT on gait speed in people with traumatic brain injury. The plot shows the effect size estimates (circles) and their confidence intervals (horizontal lines) for each study, with the overall effect size estimate (red diamond at the bottom) and its confidence interval (diamond outline) presented. The change in the overall effect size estimate after the removal of each study is shown by a black circle presented in the right-hand column.

Study name	Point	Standard error	Variance	Lower limit	Upper limit	Z-Value	p-Value
	Statistics with study removed						
Thompson et al. (2021)	0.630	0.305	0.093	0.033	1.227	2.067	0.039
Sheridan et al. (2021)	0.608	0.262	0.069	0.094	1.121	2.318	0.020
Park et al. (2015)	0.446	0.283	0.080	-0.110	1.001	1.573	0.116
Wilfong (2009)	0.306	0.260	0.067	-0.202	0.815	1.181	0.238
Hurt et al. (1998)	0.533	0.327	0.107	-0.108	1.174	1.630	0.103
Pooled	0.494	0.243	0.059	0.018	0.971	2.035	0.042

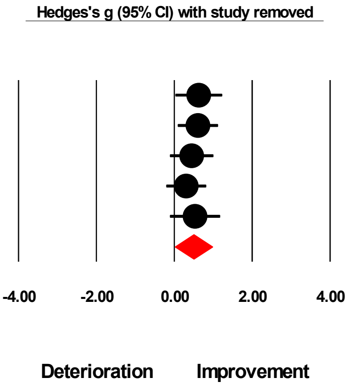

Figure S3. Forest plot depicting the results of leave-one-out sensitivity analysis for the effect of MT on cadence in people with traumatic brain injury. The plot shows the effect size estimates (circles) and their confidence intervals (horizontal lines) for each study, with the overall effect size estimate (red diamond at the bottom) and its confidence interval (diamond outline) presented. The change in the overall effect size estimate after the removal of each study is shown by a black circle presented in the right-hand column.

Study name	Subgroup within study	Point	Standard error	Variance	Lower limit	Upper limit	Z-Value	p-Value
		Statistics with study removed						
Singhal and Kataria (2021	Cadence	0.568	0.490	0.240	-0.392	1.528	1.160	0.246
Singhal and Kataria (2021	Speed	0.597	0.488	0.238	-0.360	1.553	1.222	0.222
Tamburella et al. (2021)	Speed	0.619	0.486	0.236	-0.333	1.572	1.274	0.203
Amatachaya et al. (2009)	Cadence	0.561	0.566	0.320	-0.548	1.669	0.991	0.322
Amatachaya et al. (2009)	Speed	0.135	0.157	0.025	-0.174	0.443	0.857	0.391
de l'Etoile (2008)	Cadence	0.630	0.521	0.271	-0.390	1.650	1.210	0.226
de l'Etoile (2008)	Speed	0.636	0.518	0.269	-0.381	1.652	1.226	0.220
Pooled		0.534	0.438	0.192	-0.324	1.393	1.220	0.222

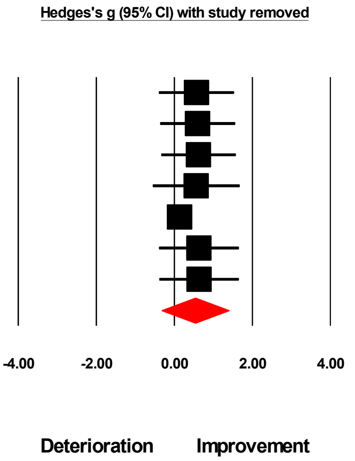

Figure S4. Forest plot depicting the results of leave-one-out sensitivity analysis for the effect of MT on overall spatiotemporal gait outcomes in people with spinal cord injury. The plot shows the effect size estimates (circles) and their confidence intervals (horizontal lines) for each study, with the overall effect size estimate (red diamond at the bottom) and its confidence interval (diamond outline) presented. The change in the overall effect size estimate after the removal of each study is shown by a black circle presented in the right-hand column.

Study name	Statistics with study removed						
	Point	Standard error	Variance	Lower limit	Upper limit	Z-Value	p-Value
Singhal and Kataria (2021)	0.971	1.086	1.179	-1.157	3.100	0.895	0.371
Tamburella et al. (2021)	1.020	1.074	1.153	-1.085	3.125	0.950	0.342
Amatachaya et al. (2009)	-0.022	0.265	0.070	-0.542	0.498	-0.082	0.935
de l'Etoile (2008)	1.045	1.103	1.216	-1.117	3.207	0.948	0.343
Pooled	0.766	0.855	0.731	-0.910	2.441	0.896	0.370

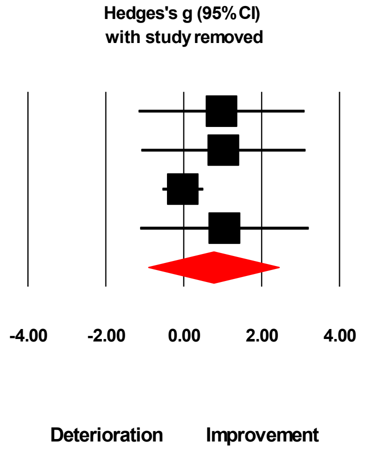

Figure S5. Forest plot depicting the results of leave-one-out sensitivity analysis for the effect of MT on gait speed in people with spinal cord injury. The plot shows the effect size estimates (circles) and their confidence intervals (horizontal lines) for each study, with the overall effect size estimate (red diamond at the bottom) and its confidence interval (diamond outline) presented. The change in the overall effect size estimate after the removal of each study is shown by a black circle presented in the right-hand column.
